# Networking State of Ytterbium Ions Probing the Origin of Luminescence Quenching and Activation in Nanocrystals

**DOI:** 10.1002/advs.202003325

**Published:** 2021-01-29

**Authors:** Sheng Mei, Jiajia Zhou, Hong‐Tao Sun, Yangjian Cai, Ling‐Dong Sun, Dayong Jin, Chun‐Hua Yan

**Affiliations:** ^1^ Institute for Biomedical Materials & Devices (IBMD) Faculty of Science University of Technology Sydney New South Wales 2007 Australia; ^2^ Beijing National Laboratory for Molecular Sciences State Key Laboratory of Rare Earth Materials Chemistry and Applications PKU‐HKU Joint Laboratory in Rare Earth Materials and Bioinorganic Chemistry College of Chemistry and Molecular Engineering Peking University Beijing 100871 China; ^3^ College of Chemistry Chemical Engineering and Materials Science Soochow University Jiangsu 215123 China; ^4^ International Center for Materials Nanoarchitectonics (MANA) National Institute for Materials Science (NIMS) Tsukuba 305‐0044 Japan; ^5^ UTS‐SUStech Joint Research Centre for Biomedical Materials & Devices Department of Biomedical Engineering Southern University of Science and Technology Shenzhen Guangdong 518055 China; ^6^ College of Chemistry and Chemical Engineering Lanzhou University Lanzhou 730000 China

**Keywords:** lanthanide nanocrystals, luminescence, quenching, ytterbium ions

## Abstract

At the organic–inorganic interface of nanocrystals, electron‐phonon coupling plays an important but intricate role in determining the diverse properties of nanomaterials. Here, it is reported that highly doping of Yb^3+^ ions within the nanocrystal host can form an energy‐migration network. The networking state Yb^3+^ shows both distinct Stark splitting peak ratios and lifetime dynamics, which allows quantitative investigations of quenching and thermal activation of luminescence, as the high‐dimensional spectroscopy signatures can be correlated to the attaching and de‐attaching status of surface molecules. By in‐situ surface characterizations, it is proved that the Yb‐O coordination associated with coordinated water molecules has significantly contributed to this reversible effect. Moreover, using this approach, the prime quencher —OH can be switched to —CH in the wet‐chemistry annealing process, resulting in the electron‐phonon coupling probability change. This study provides the molecular level insights and dynamics of the surface dark layer of luminescent nanocrystals.

## Graphical abstract

Doping Yb^3+^ ions in nanocrystals, particularly at high concentrations, enables the Yb^3+^ ions to constitute energy transfer network and display many distinct dynamic features, including the temperature‐responsive Yb^3+^ lifetimes and Stark splitting spectra. This allows us to achieve the molecular‐level understanding of the organic–inorganic interface interactions and their relations to the luminescence behaviors.

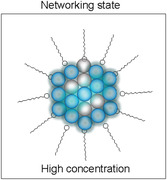

## 1 Introduction

Organic surface ligands often determine the physical and chemical properties of inorganic nanomaterials, such as facet‐dependent growth dynamics,^[^ ^1^ ^]^ colloidal stability,^[^ ^2^ ^]^ and quantum efficiency.^[^ ^3^ ^]^ For luminescent nanocrystals, the vibrating ligands, for example, oleic acid and water molecules, can interact with the inorganic nanoparticle through the —CH and —OH moiety groups, which often locally consume the excited state photon energy on the surface and quench the overall luminescence intensity through the electron‐phonon coupling.^[^ ^4^ ^]^ This is particularly true for the ultra‐small nanocrystals (∼10 nm) with a large surface‐to‐volume ratio, where the sophisticated surface interactions and quenching factors were believed to sacrifice the optically active layer into an optically deficient dark layer.^[^ ^5^, ^6^ ^]^ But the origin of this dark layer and the molecular picture of the organic–inorganic interactions at the interface remain unclear.

**Figure 1:**
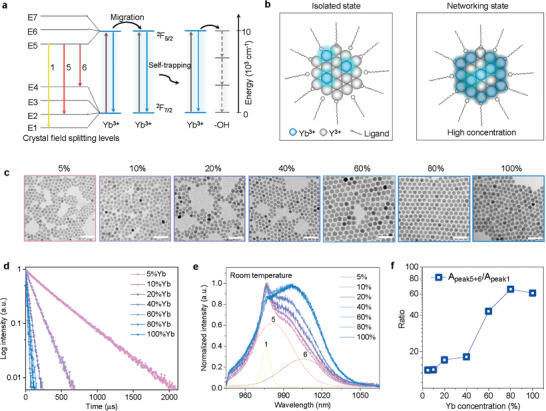
Lifetime and spectral splitting between the isolated state and the networking state of ytterbium ions, highly dependent on the doping concentrations. a) The energy level diagram of Yb^3+^ and its’ Stark splitting in a crystal field, and the energy migration, interior trapping and surface phonon coupling processes within a nanocrystal. b) Schematic illustration of the concentration‐dependent isolated and networking states of Yb^3+^ ions. c) Transmission electron microscope (TEM) images of a series of *β*‐NaYb*_x_*_%_Y_1−_ *_x_*_%_F_4_ nanocrystals, in which *x* equals to 5, 10, 20, 40, 60, 80, and 100 from left to right. Scale bar: 50 nm. d) Lifetime curves of *β*‐NaYb*_x_*_%_Y_1−_ *_x_*_%_F_4_ nanocrystals at room temperature, by the 980 nm pulsed laser excitation. e) Normalized emission spectra and f) Yb^3+^ concentration‐dependent splitting peak area ratios of *β*‐NaYb*_x_*_%_Y_1−_ *_x_*_%_F_4_ nanocrystals at room temperature, by the 915 nm continuous‐wave laser excitation.

The ytterbium (Yb^3+^) ion has a simple energy level with only the first excited electron configuration (^2^F_5/2_ level) in a narrow band of 10,000 cm^−1^. Importantly, this energy level matches very well to the sum of triple phonons of the —OH group, so that the excited state Yb^3+^ could be easily depleted by the surface —OH group via a three‐phonon relaxation process (**Figure** **1**a). Moreover, Yb^3+^ is the smallest one in the luminescent trivalent lanthanide family and has a weak screening effect of the 4f^n^ shell by the filled 5s and 5p electron shells, which further enhances the electron‐phonon interaction. As shown in Figure 1a, as long as the crystal field symmetry is lower than the cubic, the excited and ground states of Yb^3+^ ions are split into three and four Stark levels, respectively. The transitions between these splitting levels are sensitive to the lattice site environment and temperature.^[^ ^7^ ^]^ Furthermore, the narrow‐band excited state at ^2^F_5/2_ is metastable with an exceptionally long lifetime in the orders of up to a few milliseconds, which renders Yb^3+^ a high chance in “networking” with its nearby ground‐state Yb^3+^ ions to quickly migrate out its excited energy, rather than producing radiative emission. In parallel, any kind of phonon coupling of an excited state Yb^3+^ ion, either at the surface or inside the crystal, may affect the other nearby Yb^3+^ ions in a cascade fashion, so that the quenching and activation of excited state Yb^3+^ ions may be quickly amplified and displayed in the multi‐parameter Yb^3+^ spectroscopy.

Yb^3+^ ion has been widely introduced in both semiconductor and insulator crystals. Yb^3+^ doped CdSe,^[^ ^8^ ^]^ NaInS_2_,^[^ ^9^ ^]^ PbIn_2_S_4_,^[^ ^9^ ^]^ and CsPbX_3_^[^ ^10^ ^]^ nanocrystals have been successfully synthesized to facilitate the spectral conversion from ultraviolet‐visible (UV–Vis) to near‐infrared (NIR) through a quantum‐cutting process. Fluoride and oxide crystals can be co‐doped with lanthanide ions, in which Yb^3+^ serves as the photon sensitizer to either up‐convert NIR to visible and UV or downshift NIR to IR emissions.^[^ ^11^ ^]^ These nanocrystals have been widely explored towards a broad range of emerging applications spanning from bio‐imaging,^[^ ^12^ ^]^ sensing,^[^ ^13^ ^]^ nanoscopy,^[^ ^14^ ^]^ light‐triggered drug delivery,^[^ ^15^ ^]^ optogenetics,^[^ ^16^ ^]^ night vision enhancement^[^ ^17^ ^]^ to anti‐counterfeiting security inks,^[^ ^18^ ^]^ volumetric displays,^[^ ^19^ ^]^ photovoltaics,^[^ ^20^ ^]^ and photonic devices.^[^ ^21^ ^]^

High concentrations of Yb^3+^ ions above 50 mol% in the core‐shell architecture has been found to favor the high brightness of nanoparticles,^[^ ^22^ ^]^ strong UV upconversion, and lasing emission generations.^[^ ^23^ ^]^ The heterogeneous structure of pure *α*‐NaYbF_4_ active core and CaF_2_ passivation shell has been discovered to convert the pulsed NIR excitation into the long‐decaying time‐resolved emissions with quantum efficiency approaching 100%.^[^ ^24^ ^]^ Highly Yb^3+^ doped core with inert shell passivation has been demonstrated as the efficient probes with single‐particle sensitivity.^[^ ^25^ ^]^

Here, we develop a multi‐dimensional spectroscopy approach by using the highly‐doped Yb^3+^ ions (in the networking state) to probe the structural and interfacial conditions of nanocrystals. As illustrated in Figure 1b, we emphasize the large contrast in spectral physics between the low‐concentration Yb^3+^ ions (in the isolated state) and the high‐concentration scenario (in the networking state). To quantitatively investigate the concentration dependency of Yb^3+^ spectroscopy, we synthesized a series of *β*‐NaYF_4_ nanoparticles doped by the gradient concentrations of Yb^3+^ ions. TEM images in Figure 1c show the morphology of the as‐synthesized samples with Yb^3+^ doping concentrations increasing from 5 mol% to 100 mol%, in the size range of 10–16 nm (see size distributions in Figure S1, Supporting Information).

## 2 Results and Discussion

The decay curves of the Yb^3+^ excited state are shown in Figure 1d. When the doping concentrations of Yb^3+^ ions are relatively low, for example, <20 mol%, the lifetimes are rather long, which indicates a slow rate of energy migration between the isolated Yb^3+^ ions. In contrast, when the doping concentrations are further elevated from 20 mol% up to 100 mol%, samples show rather small lifetime values, around 20 µs (also see Table S1, Supporting Information). This suggests that the 20 mol% concentration is a turning point for Yb^3+^ shifting from the isolated state to its networking state. It is noteworthy that the lifetime values around 20 µs for nanocrystals with Yb^3+^ in the networking state are only a little longer than that of Yb^3+^ complexes,^[^ ^26^ ^]^ where Yb^3+^ ions are chelated directly by the organic ligands. This indicates that the Yb^3+^ ions in these nanocrystals already form a cooperative network that ‘short‐circuits’ to the Yb^3+^ ions near the surface ligands.

The steady‐state Yb^3+^ spectroscopy results, shown in Figure 1e, further reveal a remarkable redshift of the primary peak from the 978 nm to 1000 nm that broadens the full width at half maximum (FWHM), when the Yb^3+^ concentration increases, particularly above 40 mol%. We use Gaussian deconvolution fit and the 10 K emission spectrum (Figure S2, Supporting Information) to identify the three sub‐peaks that correspond with the three crystalline site splitting transitions of 1, 5, and 6 (see Figure 1a). This allows us to further quantify the redshift by the integrated intensity ratio between the peaks (5+6) and the peak 1, showing an increase from 14 to 65, sharply from the point of 40 mol% concentration (Figure 1f). By taking into the considerations of the rather constant lifetime values (Figure 1d; Table S1, Supporting Information), for samples with the concentrations above 40 mol%, the continuous increase in the peak ratio reflects the numerous steps of the energy hopping between the interior networking state Yb^3+^ ions within the lattice through a self‐trapping process.^[^ ^27^ ^]^ Here, the energy hopping process takes place with the energy gap of E5→E1 that produces the peak 1 emission. As shown in Figure S3, Supporting Information, through numerous steps of hopping within the highly‐doped nanocrystals, the transition energy of peak 1 emission has a high chance to be quenched by the surface induced phonon coupling, while the energy hopping between E5→E2 and E5→E4, producing emission peaks 5 and 6, are unlikely to be quenched by the surface phonon coupling. Therefore, this dynamic difference of peaks (5+6) and peak 1 in the networking state results in the intensity ratio changes.

**Figure 2:**
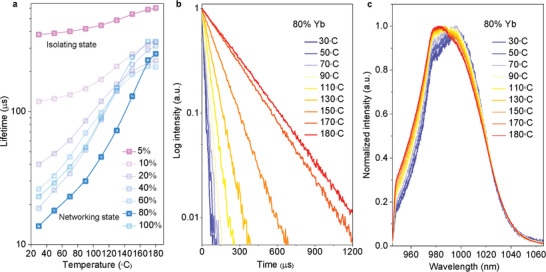
Spectroscopic signature of networking state Yb^3+^ at elevated temperatures, showing the spectroscopy probing capability of Yb^3+^ in revealing the luminescence quenching and activation behaviors. a) Evolution of luminescence decay lifetime values for *β*‐NaYb*_x_*_%_Y_1−_ *_x_*_%_F_4_ nanocrystals in the thermal field of elevated temperatures. b) Decay curves of NaYF_4_:80 mol% Yb^3+^ nanocrystals at the elevated temperatures, by 980 nm pulsed laser excitation. c) Normalized emission spectra of NaYF_4_:80%Yb^3+^ nanocrystals at the elevated temperatures, by 915 nm continuous‐wave laser excitation.

Next, we applied the thermal field by increasing the surrounding temperatures to measure the same series of samples with the gradient Yb^3+^ concentrations. The TEM images for the sample before and after treatment (Figure S4, Supporting Information) confirmed the morphological stability of NaYF_4_:Yb^3+^ nanocrystals during the heating process. The inductively coupled plasma–atomic emission spectrometry (ICP‐AES) analyses (Table S2, Supporting Information) combined with NIR emission spectra (Figure S5, Supporting Information) confirmed the absence of impurity ions such as Er^3+^ in the samples. As shown in **Figure** **2**a, Figures S6 and S7 and Table S1, Supporting Information, we observed both the remarkably prolonged lifetime values and enhanced emission intensities for all the samples, suggesting the temperature‐induced activation of the optically silent Yb^3+^ ions.^[^ ^6^ ^]^ Again, remarkably, the slopes of the enhancement factors are highly concentration‐dependent, where there are sharp rises for the samples with the networking state Yb^3+^ ions. As shown in Figure 2b, the lifetime of NaYF_4_:80%Yb^3+^ sample has been prolonged by more than one order of magnitude, from 14 µs to 272 µs. The blue‐shift of emission spectra at the elevated temperatures (Figure 2c) further implies that high temperatures can reduce the degree of non‐radiative energy down‐shift between the networking state Yb^3+^ ions, though the ratio of peak (5+6)/peak 1 at 180 °C for the high‐concentration samples remained higher than that for the NaYF_4_:5%Yb^3+^ sample (Figure S8, Supporting Information). This suggests that at high temperatures the interior energy hopping between the networking state Yb^3+^ ions remain prominent. Moreover, the spectra broadening is positively correlated with the amount of certain quenching factors on the surface, as the nanocrystals with inert shell protection can fully suppress the variation of splitting peak ratio.^[^ ^24^ ^]^ Therefore, from the above analysis, we can conclude that nanocrystals with 80 mol% Yb^3+^ can well represent the networking state Yb^3+^ spectroscopic features in both splitting peak and lifetime changes and can be used to probe the surface‐induced quenching and temperature‐induced activation behaviors of nanocrystals.

**Figure 3:**
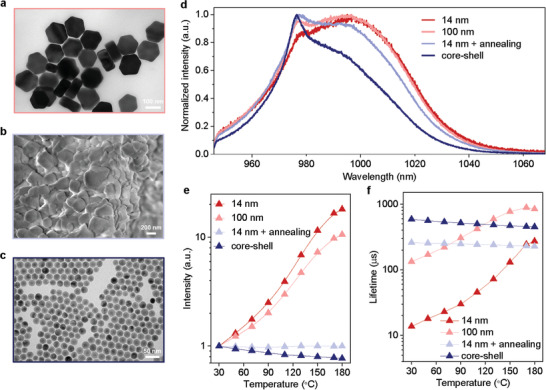
The networking state ytterbium spectroscopy reveals the surface and interior status of nanomaterials. a) TEM images of NaYF_4_:80%Yb nanomaterials with large size of 100 nm diameter, and b) 450 °C annealing in Argon atmosphere, and c) passivation shell coating of the 14 nm nanocrystals. d) Normalized emission spectra of NaYF_4_:80%Yb samples at room temperature by the 915 nm continuous‐wave laser excitation. e) Temperature‐dependent normalized luminescence intensity and f) lifetime change for the NaYF_4_:80%Yb samples.

To demonstrate the power of using the networking state Yb^3+^ to probe the surface and interior status of nanomaterials, and based on the 14 nm NaYF_4_:80%Yb^3+^ nanocrystals, we further prepared a series of 80 mol%Yb^3+^ samples. These include 100 nm crystals (**Figure** **3**a) to reduce the surface to volume ratio by around seven times, nanomaterials of the original 14 nm nanocrystals after 450 °C annealing in Argon atmosphere (Figure 3b) to dramatically reduce the amount of organic quenching factors, and ∼5.5 nm inert shell coating (NaYF_4_:80%Yb@NaYF_4_) (Figure 3c) to passivate the active core from the surface quenchers. From the room temperature emission spectra shown in Figure 3d, the slight difference between 100 nm sample and 14 nm sample suggests the effective energy hopping between the networking‐state Yb^3+^ ions regardless of the location of Yb^3+^ ions within the crystal matrix. The spectrum profile for nanomaterials after the annealing process, compared with the core‐shell samples, suggests the increased steps and longer distance of hopping process between the interior networking‐state Yb^3+^ ions within the lattice of the rather large and aggregated form of the materials. The probing fidelity could be further checked by the structural information data, in which the surface molecules measured by Fourier transform infrared spectroscopy (FTIR) for the 14 nm, 100 nm, and annealing samples are presented in the descending order (Figure S9, Supporting Information). The ∼5.5 nm inert shell leads to the negligible degree of surface quenching, though the core‐shell sample has similar surface ligand content as the 14 nm sample (Figure S10, Supporting Information). Through monitoring the temperature‐dependency changes in emission intensity (Figure 3e) and lifetime (Figure 3f), the degrees of temperature‐induced activations of Yb^3+^ luminescence can be highly correlated to the amounts of surface phonon coupling and quenchers. The difference between the annealed and core‐shell samples indicates the existence of interior thermal quenching factors rather than the surface molecules.

**Figure 4:**
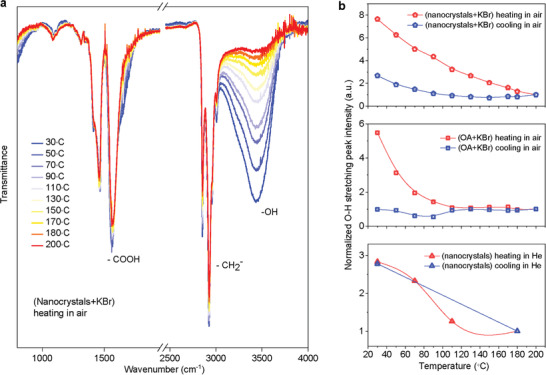
In‐situ FTIR characterization reveals the coordination role of water molecules at the surface of nanocrystals. a) FTIR spectra of NaYF_4_:80%Yb^3+^ nanocrystals (14 nm) during the heating process, which was measured in air by mixing the powder state particles with KBr. b) The —OH stretching intensity evolution during the heating and cooling cycle of the powder state NaYF_4_:80%Yb^3+^nanocrystals (14 nm) and KBr mixture measured in air (top), oleic acid and KBr mixture measured in air (middle), and pure powder state NaYF_4_:80%Yb^3+^ nanocrystals (14 nm) in helium (bottom).

To further obtain the molecular level insight on how the surface species, for example, OA and water, affect the electronic transitions and how they effectively activate the quenched luminescence in a thermal field, we conducted a series of in‐situ surface and structural characterizations for the 14 nm NaYF_4_:80%Yb nanocrystals. We first investigated FTIR by increasing the temperature to check the responsive variations of vibration strength (**Figure** **4**a). We found that only the vibration strength of hydroxyl group (—OH) at 3413 cm^−1^ decreased monotonically with temperature increase, while the other vibrations from oleate anions, including carboxylic stretching (—COO^−^) at 1465 and 1560 cm^−1^, methylene stretching (—CH_2_) at 2854 and 2925 cm^−1^, displayed negligible changes. This indicates that it might be the water molecule that dominates the temperature‐dependent Yb^3+^ spectroscopy results. Moreover, by monitoring the FTIR spectra during the cooling cycle (Figure 4b top; Figure S11, Supporting Information), we observed a partial recovery of the vibration strength of (—OH). Intriguingly, this recovery behavior disappeared in the control sample of OA and KBr powder (Figure 4b middle; Figure S12, Supporting Information), in which the (—OH) group was from the free water absorbed by KBr. This rules out the effect caused by the free water molecules in the process of the (—OH) vibration recovery during the cooling cycle. We then removed the absorbed free water molecules in the powder state nanocrystals via the process of helium gas blowing and sample sealing, and consequently measured the in‐situ infrared diffuse reflectance spectroscopy to evaluate the temperature‐dependent (—OH) vibration behavior (Figure S13, Supporting Information). As shown in Figure 4b (bottom), the vibration strength of the (—OH) group in nanocrystals was fully recovered during the entire cycle of heating and cooling. These results imply a relatively strong binding force between the water molecules and nanocrystals, and most probably in the coordination form between the cation Yb^3+^ and water molecules.

**Figure 5:**
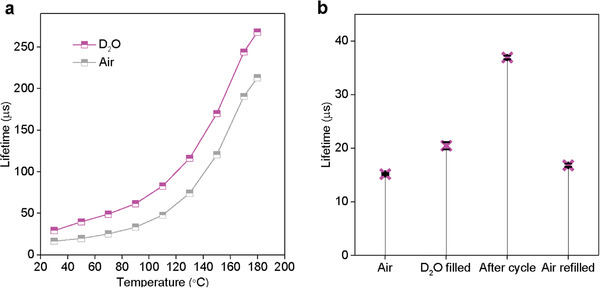
Heavy water replacement experiment confirms the displacement dynamic role of water molecules at the nanoparticle surface. a) Comparison of temperature‐dependent Yb^3+^ lifetime values in 14 nm NaYF_4_:80%Yb^3+^ nanocrystals during the heating process in the presence (magenta) and absence (grey) of D_2_O atmosphere. b) Room temperature lifetime values of 14 nm NaYF_4_:80%Yb^3+^ nanocrystals in the air, in the presence of D_2_O before heating, after heating, and leaving the D_2_O replaced sample in air overnight.

As the quenching effect of (—OD) group vibration could be much weaker than that of (—OH) due to the larger atomic mass,^[^ ^28^ ^]^ we intentionally added ≈0.5 g D_2_O into the powder state nanocrystals in a closed chamber and monitored the changes in the temperature‐dependent lifetime values of Yb^3+^. As shown in **Figure** **5**a and Figure S14 as well as Table S3, Supporting information, the lifetime values in D_2_O are always larger than that in air, which implies the replacement of H_2_O by D_2_O and the coordination between (‐OD) and Yb^3+^ formed at the surface of nanocrystals. As the dynamic exchange process reaches the equilibrium state after a heating and cooling cycle, increased number of (‐OD) and Yb^3+^ binding at the surface of nanocrystals resulted in the longest room temperature lifetime (Figure 5b), compared with other conditions including the sample in the air, filling D_2_O before heating, and leaving the exchanged sample in the air overnight. These results further confirm the temperature‐dependent luminescent properties of nanocrystals and the coordination/displacement dynamics of the coordinated water molecules on the nanocrystals.

**Figure 6:**
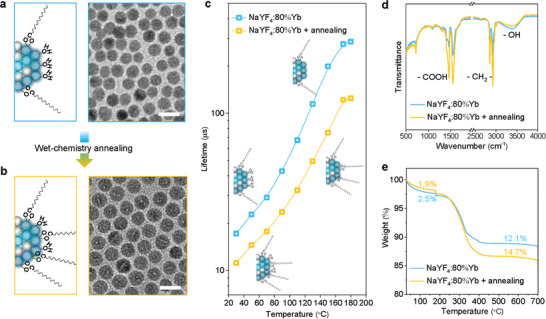
Apply networking state spectroscopy to understand the wet‐chemistry annealing effect on luminescence. Schematic illustration of the surface difference of a nanocrystal a) before and b) after wet‐chemistry annealing in the hot OA and ODE solvents. TEM images of NaYF_4_:80%Yb^3+^nanocrystals (a) before and (b) after wet‐chemistry annealing. Scale bar, 20 nm. c) Comparison of temperature‐dependent lifetime values of the powder state samples before and after wet‐chemistry annealing. d) Room temperature FTIR spectra and e) thermal gravimetric analysis (TGA) of the powder state samples before and after wet‐chemistry annealing.

The networking state Yb^3+^ spectroscopy provides new insights in energy migration, interior trapping, the origins of surface quenching, and the dynamics of temperature‐induced luminescence activation, especially at the intricate nanoscale interface. Here, we apply this approach to examine the results in the structure‐optical properties of nanocrystals post‐treated by a recently reported method of wet‐chemistry annealing.^[^ ^29^, ^30^ ^]^ The wet‐chemistry annealing has been reported to be effective in reducing deleterious defects on the surface of KLu_2_F_7_:Yb^3+^, Er^3+^ nanocrystals, which led to more than tenfold enhancement of emission intensity as compared to the primary sample,^[^ ^29^ ^]^ while obvious elemental migration during the annealing has been reasoned for the observed decreased emission intensity in NaErF_4_@NaYF_4_ nanocrystals.^[^ ^30^ ^]^ We believe that the surface of nanocrystals led to the seemingly inconsistent phenomenological results in the different energy transfer systems. We infer that the ligand species of oleate and coordinated water molecules as well as their ratios at the surface of nanocrystals will change dramatically during the treatment, as illustrated in **Figure** **6**a,b, and therefore the enhanced or quenched luminescence may occur depending on the degree in the coupling efficiency between surface molecule and excited state lanthanides.

We annealed the NaYF_4_:80%Yb^3+^ nanocrystals in hot OA and 1‐octadecene (ODE) solvents. As the TEM images shown in Figure 6a,b and their size distributions in Figure S15, Supporting Information shows, no obvious morphology change of nanocrystals were observed. Strikingly, the temperature‐dependent lifetime values of Yb^3+^ displayed a remarkable difference. As shown in Figure 6c and Figure S16 as well as in Table S4, Supporting Information, the Yb^3+^ lifetime values for the wet‐chemistry annealed nanocrystals were always lower than those of the primary nanocrystals under the same temperature, and the discrepancy became larger as the temperature increased higher. We measured the infrared spectra of the two samples using attenuated total reflectance FTIR (ATR‐FTIR) at room temperature (Figure 6d). From the intensity ratio of —OH vibration and —CH vibration, we can see that a higher (—CH_2_^−^)/(—OH) ratio exists in the wet‐chemistry annealed nanocrystals, as illustrated in Figure 6a. This has been further verified by thermogravimetric analysis (TGA) results in Figure 6e. Under the same heating procedure, more mass loss happened in primary nanocrystals below 180 °C, which mainly came from the desorption of coordinated water molecules (2.5% vs. 1.9%). When elevating the temperature to 700 °C, the overall mass loss of annealed nanocrystals became larger, which implies a higher mass percentage of OA^−^ ligands. Taking the quenching effects of —OH and —CH into the considerations, the difference in (‐CH_2_^−^)/(‐OH) ratios could well account for the differences in temperature‐dependent lifetime values. At room temperature, vibrations from —OH and —CH, in which —OH vibrations played a prime role due to stronger quenching efficiency, heavily quenched the luminescence of Yb^3+^ in both samples. As a result, lifetime values in both samples were very short. The quenching induced by ‐OH can be gradually alleviated due to the displacement of coordinated water molecules with the temperature increase, and the quenching effect of —CH vibrations becomes magnified and dominant under high temperature (≈180°C). Therefore, the gap in lifetime values between the two samples with different amounts of surface ligands enlarges at the elevated temperatures. Our results, obtained from networking state Yb^3+^ spectroscopy and verified by FTIR and TGA measurements, reveal a transition of the dominant quencher from —OH to —CH in the wet‐chemistry annealing process.

## 3 Conclusion

In conclusion, doping Yb^3+^ ions in nanocrystals, particularly at high concentrations, has enabled the Yb^3+^ ions to network with each other and display many distinct dynamic features, including the temperature‐responsive Yb^3+^ lifetimes and Stark splitting spectra. This series of high‐dimensional spectroscopy information can be used to probe the status of surface quenching and temperature activation. This allows us to achieve the molecular‐level understanding of the organic–inorganic interface interactions and their relations to the luminescence behaviors. The Stark splitting spectra can be more associated to the multiple steps of energy hopping between interior Yb^3+^ ions, while the lifetime values more reflect the degrees of surface quenching, and the temperature‐dependency shows the dynamics of thermal activation of luminescence as a result of the displacement of the coordinated water molecules. Using this approach, in conjugation with conventional in‐situ characterization techniques, we can examine the surface molecular conditions, the quality of nanocrystals, as well as the luminescence quenching and enhancement factors at the nanoscale interface.

## 4 Experimental Section

##### Chemicals

YCl_3_·6H_2_O (99.99%), YbCl_3_·6H_2_O (99.99%), NaOH (>98%), NH_4_F (>98%), OA (90%), and ODE (>90%) were purchased from Sigma‐Aldrich. Ethanol (AR), methanol (AR), and cyclohexane (AR) were purchased from Chem‐Supply Pty. Ltd. All chemicals were used as received without further purification.

##### Synthesis of ∼10 nm *β*‐NaYF_4_:Yb^3+^ Nanocrystals

Small *β*‐NaYF_4_:Yb^3+^ nanocrystals with different Yb^3+^ doping ratios were all prepared following a modified co‐precipitation method. Typically, a given amount of RECl_3_·6H_2_O (RE^3+^ = Y^3+^/Yb^3+^, 1 mmol) was mixed with 6 mL OA and 15 mL ODE in a three‐necked flask (50 mL) at room temperature. The slurry was heated to 150 °C for 30 min with vigorous magnetic stirring under Ar atmosphere and then cooled down to room temperature. Methanol solution dissolved with 4 mmol NaOH was added into the flask with stirring for 30 min. The slurry was heated to 100 °C for 30 min to remove methanol and was cooled to room temperature again. 8 mL methanol solution of 4 mmol NH_4_F was added to the flask followed by 30 min stirring. The slurry was heated to 100 °C for 30 min to remove methanol and was further heated to 300 °C and kept for 45 min under Ar atmosphere. After cooling to room temperature, the nanoparticles were precipitated by centrifugation after adding an excess amount of ethanol. The nanoparticles were further washed with cyclohexane/ethanol for three times. The final nanocrystals were dispersed in cyclohexane or dried at 60 °C for use.

##### Synthesis of ∼100 nm *β*‐NaYF_4_:80%Yb^3+^ Nanocrystals

Large *β*‐NaYF_4_:80%Yb^3+^ nanocrystals were prepared by the traditional co‐precipitation method. The mixture of 0.8 mmol YbCl_3_·6H_2_O, 0.2 mmol YCl_3_·6H_2_O, 6 mL OA, and 15 mL ODE were heated to 150 °C for 30 min with vigorous magnetic stirring under Ar atmosphere and then cooled down to room temperature. Methanol solution dissolved with 2.5 mmol NaOH and 4 mmol NH_4_F was add to the flask followed by 30 min stirring. The slurry was heated to 100 °C for 30 min to remove methanol and was further heated to 300 °C and kept for 90 min under Ar atmosphere. The nanocrystals were precipitated and washed following with cyclohexane/ethanol for three times.

##### Synthesis of Core‐Shell Nanocrystals

Core‐shell nanocrystals were prepared by the hot injection method, which involves the preparation of shell precursor. 1 mmol YCl_3_·6H_2_O was mixed with 6 mL OA and 15 mL ODE in a three‐necked flask (50 mL) and heated to 150 °C for 30 min with vigorous magnetic stirring under Ar atmosphere and then cooled down to room temperature. Methanol solution dissolved with 2.5 mmol NaOH and 4 mmol NH_4_F was add to the flask followed by 30 min stirring. The slurry was heated to 100 °C for 30 min to remove methanol and cooled down to room temperature to get the shell precursor solution. For the growth of shell layer, cyclohexane solution containing previously prepared 0.2 mmol *β*‐NaYF_4_:80%Yb^3+^ nanocrystals were mixed with 3.6 mL OA and 9 mL ODE in a three‐necked flask (50 mL) and heated to 100 °C for 30 min with vigorous magnetic stirring under Ar atmosphere to remove cyclohexane. The mixture solution was heated to 300 °C and 0.2 mL shell precursor solution was injected every 2 min using a syringe. After the reaction, the core/shell nanocrystals were precipitated and washed with cyclohexane/ethanol for three times.

##### Heat‐Treatment of *β*‐NaYF_4_:80%Yb^3+^

The dried nanocrystals were added into a quartz crucible and put in a furnace. The sample was heated to 450 °C and kept for 60 min under Ar atmosphere.

##### Wet‐Chemistry Annealing of *β*‐NaYF_4_:80%Yb^3+^ Nanocrystals

Cyclohexane solution containing 0.2 mmol previously prepared *β*‐NaYF_4_:80%Yb^3+^ nanocrystals, 3.6 mL OA, and 9 mL ODE were mixed and heated to 100 °C for 30 min to remove cyclohexane. The solution was further heated to 300 °C and maintained for 60 min. After the reaction, the core‐shell nanocrystals were precipitated and washed with cyclohexane/ethanol for three times.

##### TEM Characterization

The morphology characterization of the nanocrystals was performed by transmission electron microscope (TEM), JEOL TEM‐1400 at an acceleration voltage of 120 kV.

##### Optical Characterization

The emission spectra of Yb^3+^‐doped samples were recorded using a home‐built spectroscopic system. A 915 nm continuous‐wave diode laser was used as the excitation source, which passed through an objective lens, and then was focused on a size‐controllable spot covering the powder sample. The dried powder samples were located in a copper holder, which was mounted on an XYZ stage to ensure the appropriate position. The temperature of the sample was controlled by a heating system assembled with a metal‐ceramic heater (HT24S2, Thorlabs) and a heater controller (TC200‐EC, Thorlabs). Emission spectra were collected by another objective lens after passing through a 937 nm long‐pass filter and then detected by a spectrometer (iHR550, Horiba).

The D_2_O filling control was completed in a heating system equipped with an enclosed heating chamber (HSF600E‐P, Linkam) and a heater controller (TMS 94, Linkam). D_2_O was added into the chamber before the heating and measurement.

The lifetime of Yb^3+^‐doped samples was measured through a time‐gating mode. We modulated a 976 nm diode laser to produce 2000 µs excitation pulses. The emission signal was collected at 980 nm through a 50 µm fiber. The luminescence was collected by a single‐photon counting avalanche photodiode (SPAD, SPCM‐AQR‐14‐FC, Excelitas Inc.) with 1000 cycles and 20 µs gating time. The pulsed excitation and collection are controlled and synchronized using a multifunction data acquisition device (USB‐6343, National Instruments) and a purpose‐built LabVIEW program.

10 K emission spectra of NaYF_4_:80%Yb^3+^ nanocrystals under 930 nm laser excitation was obtained using iHR550 spectrometer equipped with a photomultiplier tube (Hamamatsu, H10330‐75). The sample was cooled by a closed‐cycle He cryostat.

##### Infrared Spectra Measurements

FTIR spectra during the heating and cooling process were conducted on an infrared spectrometer (Nicolet 6700, Thermo Fisher) connected with a heater and controller. Samples were firstly mixed and ground with KBr pellets and pressed into tablets for in‐situ measurements.

Infrared diffuse reflectance spectroscopy was conducted using an infrared spectrometer (TENSOR 27, Bruker). Powder sample was added into the in‐situ cell (Praying Mantis, Harrick Scientific) in Helium atmosphere for measurements.

ATR‐FTIR spectra of the nanocrystals were obtained using an infrared spectrometer (Nicolet 7650, Thermo Fisher) with diffuse reflectance sampling accessories.

##### TGA Analysis

TGA was performed on a SQ600 (TA Instruments). The samples were treated with the same procedure: heating from 30 °C to 180 °C at the rate of 5 °C min^−1^; isothermal measurement at 180 °C for 30 min; heating from 180 °C to 700 °C at the rate of 10 °C min^−1^; isothermal measurement at 700 °C for 30 min.

##### Statistical Analysis

The maximum intensity was normalized to 1 in the comparison of Yb^3+^ concentration‐dependent splitting peak area ratios of *β*‐NaYb*_x_*_%_Y_1−_ *_x_*_%_F_4_ nanocrystals at room temperature. The maximum emission intensity of different samples under 30 °C was also normalized to 1 for the comparison of temperature‐dependent luminescence intensity. The —OH stretching intensity under 180 °C was normalized to 1 when comparing the intensity evolution during the heating and cooling cycle. More than 100 nanocrystals were measured to get the mean size and size distribution for each sample. Decay curve for each sample and each temperature point was measured at least three times to ensure a steady value, and the decay curve was fitted using single exponential decay function through the OriginPro 2016 software.

## Conflict of Interest

The authors declare no conflict of interest.

## Supporting information

Supporting Information
