# Supplementary material for: Networking State of Ytterbium Ions Probing the Origin of Luminescence Quenching and Activation in Nanocrystals
Source: Adv Sci (Weinh). 2021 Jan 29;8(6):2003325. doi: 10.1002/advs.202003325 (PMC7967042; doi:10.1002/advs.202003325)
Supplement: Supplementary file 1 — Supporting Information [file ADVS-8-2003325-s001.pdf]

WILEY-VCH

© 2020 Wiley-VCH GmbH

ADVANCED SCIENCE
Open Access

Supporting Information

for *Adv. Sci.,* DOI: 10.1002/advs.202003325

Networking State of Ytterbium Ions Probing the Origin of Luminescence Quenching and Activation in Nanocrystals

Sheng Mei, Jiajia Zhou, Hong-Tao Sun, Yangjian Cai, Ling-Dong Sun, Dayong Jin*,Chun-Hua Yan**

Supporting Information

Networking State of Ytterbium Ions Probing the Origin of Luminescence Quenching and Activation in Nanocrystals

Sheng Mei, Jiajia Zhou, Hong-Tao Sun, Yangjian Cai, Ling-Dong Sun, Dayong Jin*, Chun-Hua Yan**

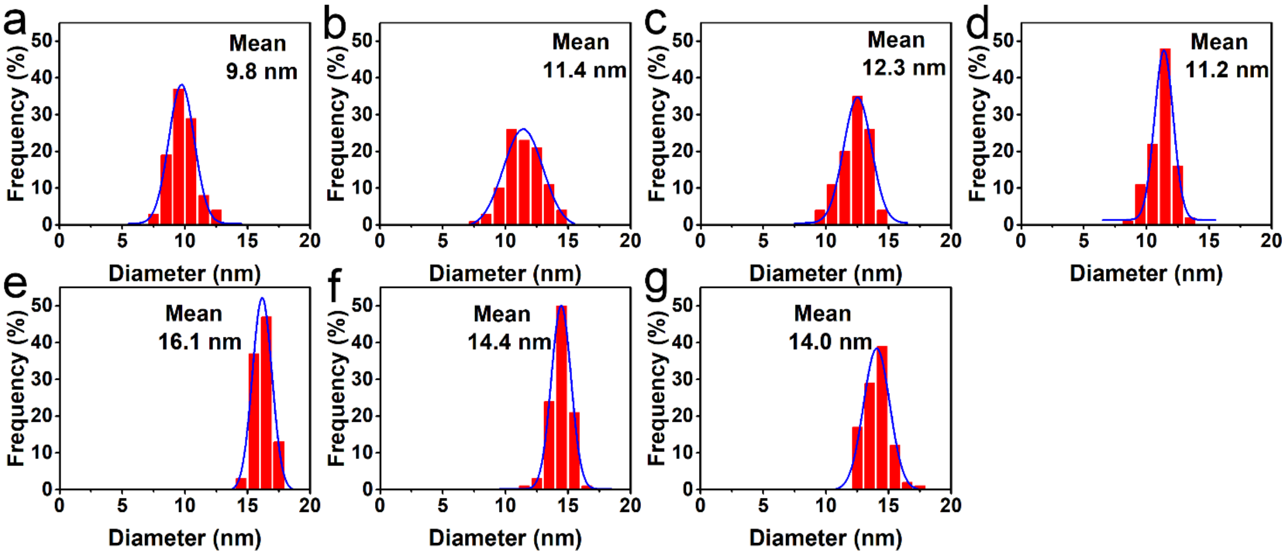

Figure S1. Size distributions of β-$NaYb_{x\%}Y_{1-x\%}F_4$ nanocrystals: (a) x = 5, (b) x = 10, (c) x = 20, (d) x = 40, (e) x = 60, (f) x = 80, (g) x = 100.

Table S1. Lifetime values of β-$NaYb_{x\%}Y_{1-x\%}F_4$ nanocrystals under elevated temperatures.

β-$NaYb_{x\%}Y_{1-x\%}F_4$	x = 5	x = 10	x = 20	x = 40	x = 60	x = 80	x = 100
30°C (μs)	379.6	119.2	40.1	18.7	26.1	13.7	22.9
50°C (μs)	387.9	124.9	48.3	25.6	32.8	18.0	29.0
70°C (μs)	401.1	133.6	60.5	34.1	45.5	22.9	38.2
90°C (μs)	420.1	148.4	85.0	51.2	68.9	29.9	53.7

110°C (μs)	445.4	174.2	119.4	86.7	98.2	45.5	89.6
130°C (μs)	486.2	218.4	160.5	143.4	143.0	71.9	151.4
150°C (μs)	541.4	271.0	202.2	225.6	190.9	130.3	257.3
170°C (μs)	585.4	318.3	231.9	289.7	219.1	243.9	336.3
180°C (μs)	601.8	333.3	241.9	311.5	216.2	271.9	335.4

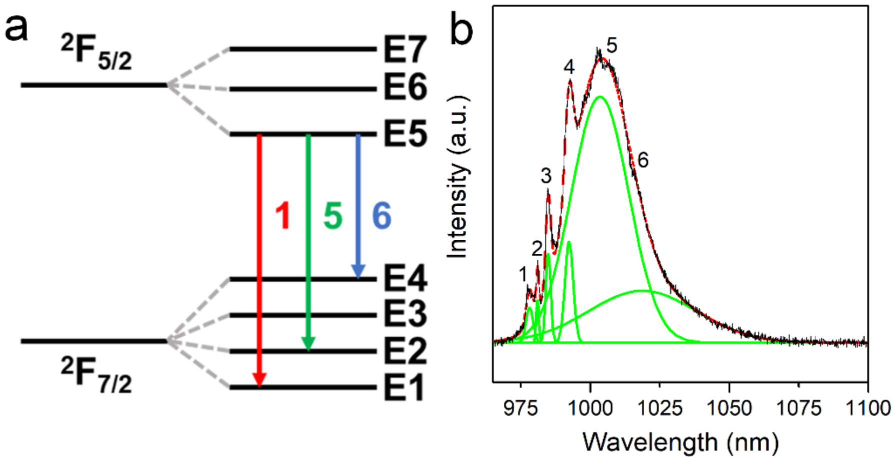

Figure S2. (a) Crystal field energy level configuration of Yb^{3+} in β-$NaYF_4$ structure, (b) 10K emission spectra of $NaYF_4$:80%Yb nanocrystals under 930 nm laser excitation.

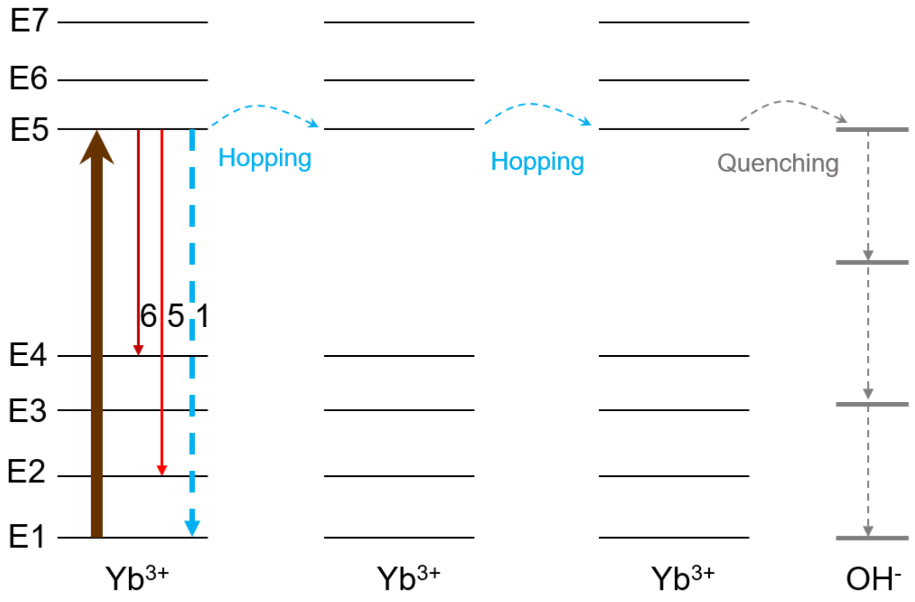

Figure S3. Schematic illustration of energy hopping process in high-doping Yb^{3+} nanocrystals.

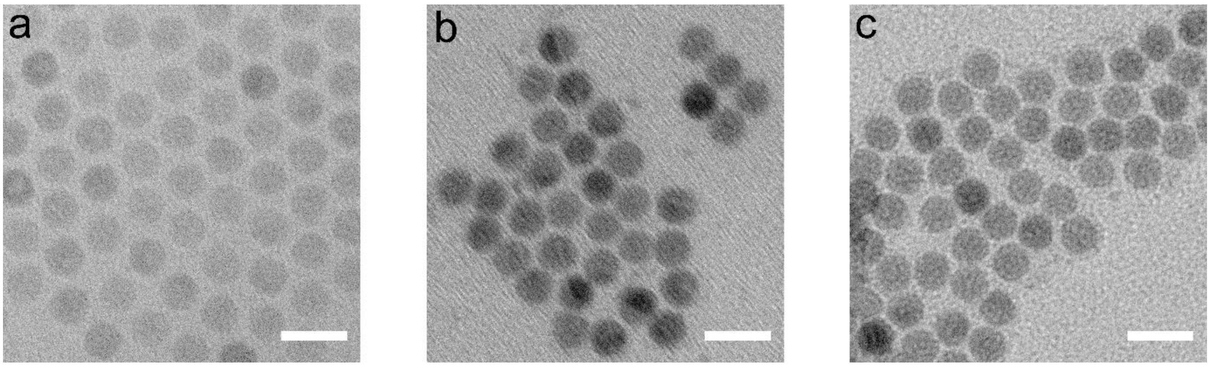

Figure S4. TEM images of β-$NaYF_4$:80%Yb^{3+} nanocrystals: (a) as-synthesized nanocrystals without treatment, (b) dried powder before heating, (c) dried powder after the heating and cooling cycle. The scale bar is 20 nm.

Table S2. ICP-AES analyses of typical β-$NaYF_4$:80%Yb^{3+} nanocrystals

Weight of sample (g)	Volume (mL)	Element	Concentration (mg/L)	Dilution	Mass Percentage	Molar Percentage
0.0232	10	Y	1.31	100	5.64%	18.2%
0.0232	10	Yb	11.45	100	49.36%	81.8%
0.0232	10	Er	0.04	1	0.00%	0.0%

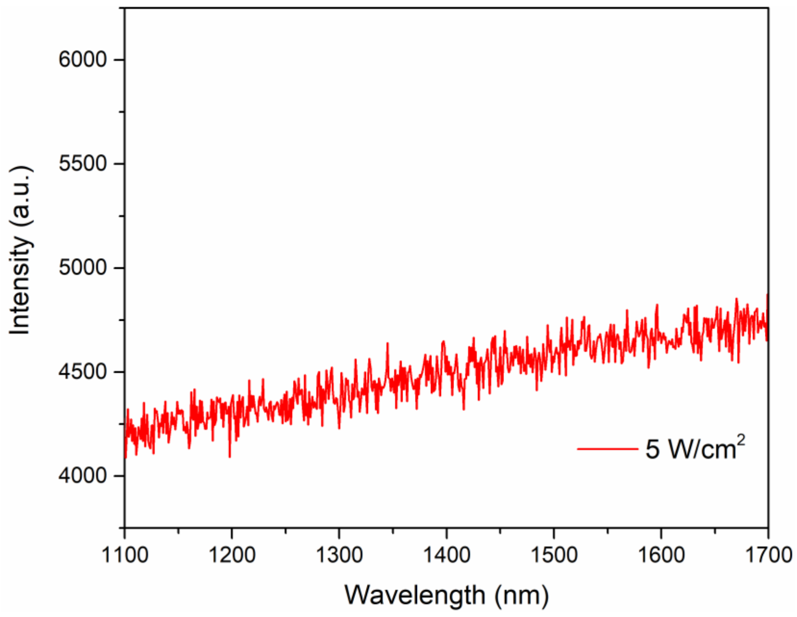

Figure S5. NIR emission spectrum from β-$NaYF_4$:80%Yb^{3+} nanocrystals under 980 nm laser excitation. This confirms the absence of impurity ion based emission such as Er^{3+}.

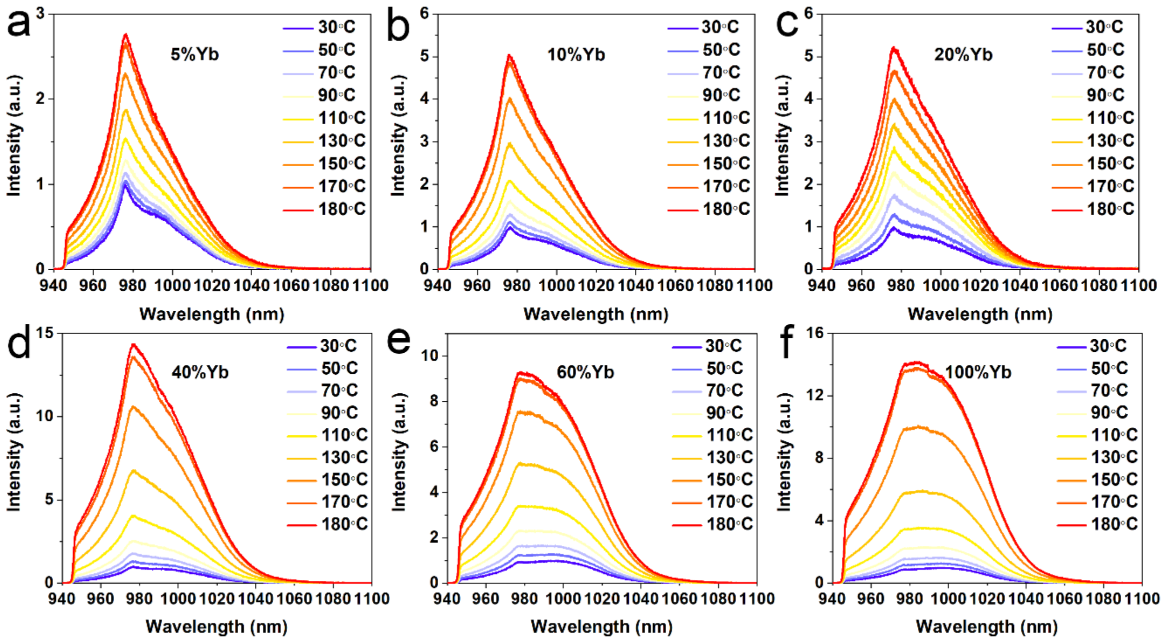

Figure S6. Evolution of emission spectra of β-NaYb$_{x\%}$Y$_{1\text{-}x\%}$F$_4$ nanocrystals under elevated temperatures. (a) $x = 5$, (b) $x = 10$, (c) $x = 20$, (d) $x = 40$, (e) $x = 60$, (f) $x = 100$.

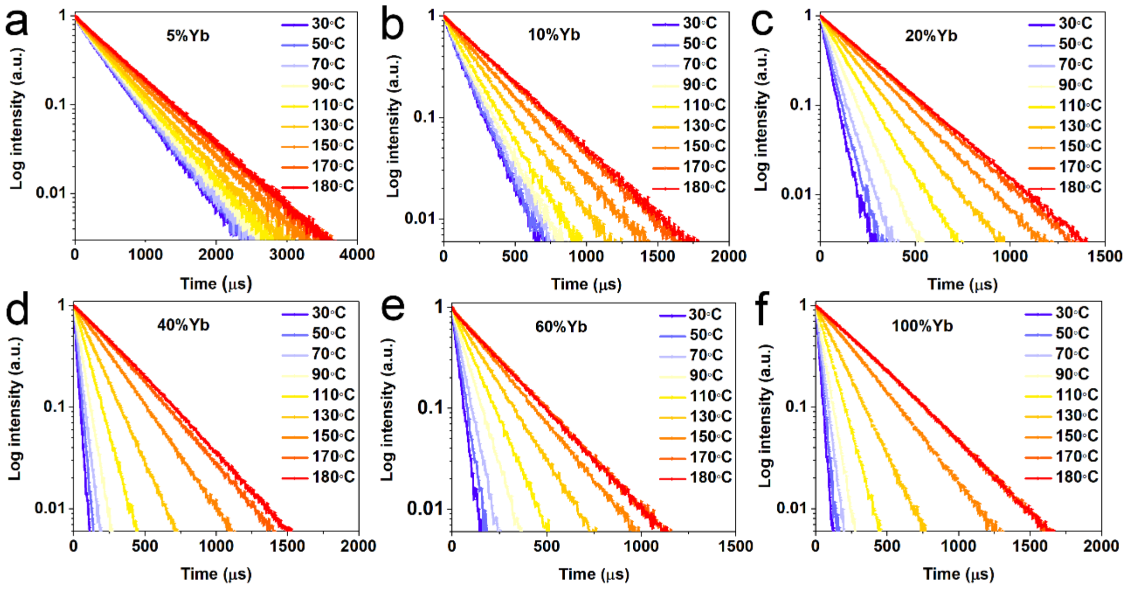

Figure S7. Evolution of lifetime curves under elevated temperatures in β-NaYb$_{x\%}$Y$_{1\text{-}x\%}$F$_4$ nanocrystals. (a) $x = 5$, (b) $x = 10$, (c) $x = 20$, (d) $x = 40$, (e) $x = 60$, (f) $x = 100$.

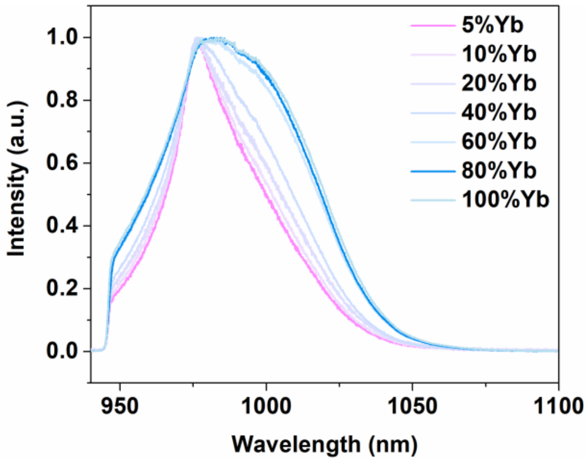

Figure S8. Normalized emission spectra of β-$NaYb_{x\%}Y_{1-x\%}F_4$ nanocrystals under 180°C.

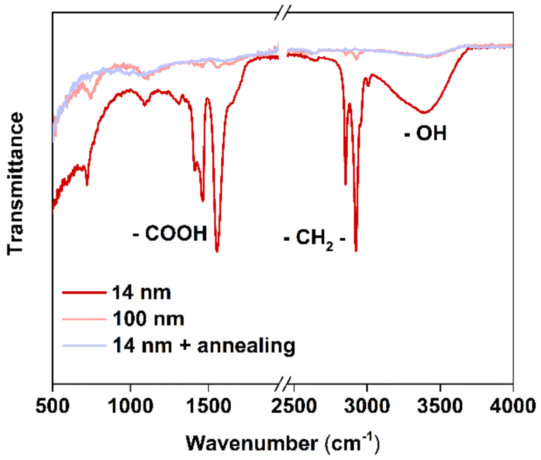

Figure S9. FTIR spectra of 14 nm, 100 nm and annealed $NaYF_4$:80%Yb samples under 30 °C.

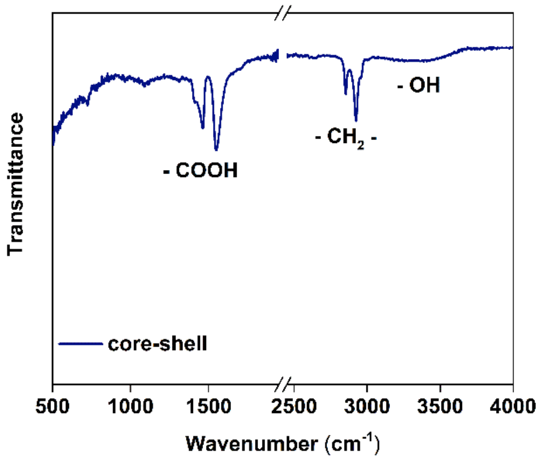

Figure S10. FTIR spectra of 25 nm $NaYF_4$:80%Yb@$NaYF_4$ core-shell nanocrystals under 30 °C.

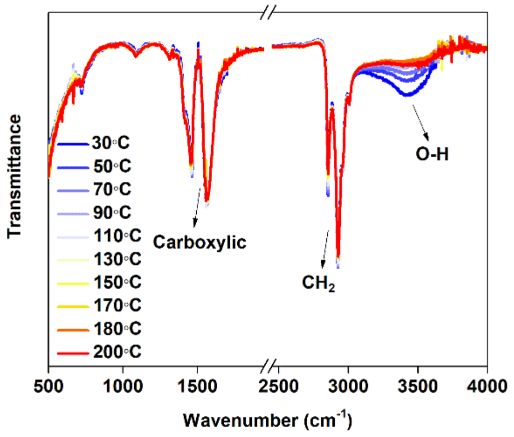

Figure S11. FTIR spectra of $NaYF_4$:80%Yb nanocrystals during the cooling process.

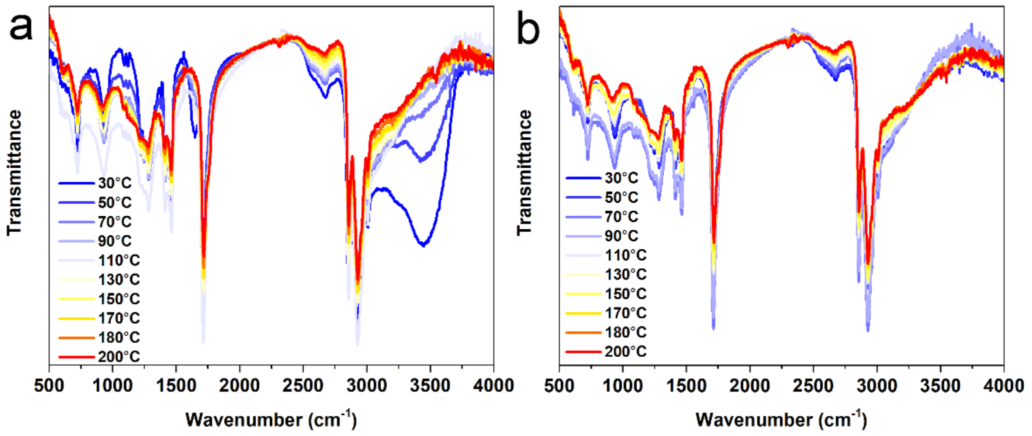

Figure S12. FTIR spectra of oleic acid during the (a) heating and (b) cooling process. Oleic acid was mixed with KBr for measurement.

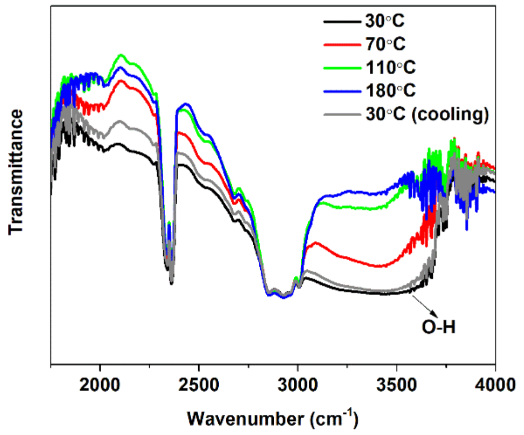

Figure S13. Infrared diffuse reflectance spectroscopy of $NaYF_4$:80%Yb nanocrystals in Helium atmosphere during the heating and cooling process.

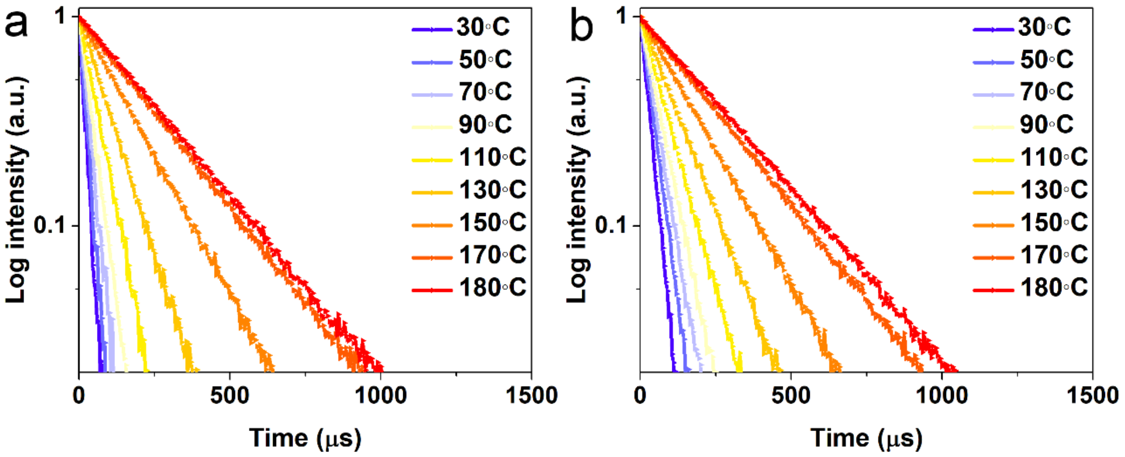

Figure S14. Lifetime curves of $NaYF_4$:80%Yb nanocrystals under elevated temperatures measured in a closed Linkam chamber filled with (a) air, (b) air and ~0.5g D_2O.

Table S3. Lifetime value comparison of $NaYF_4$:80%Yb nanocrystals under elevated temperatures in a closed Linkam chamber with a different atmosphere.

Temperature	30°C	50°C	70°C	90°C	110°C	130°C	150°C	170°C	180°C
In air (μs)	16.5	19.7	25.2	33.2	47.7	74.1	120.3	190.6	212.9
air & D_2O (μs)	29.2	39.7	49.1	61.5	82.8	116.1	170.	243.9	267.9

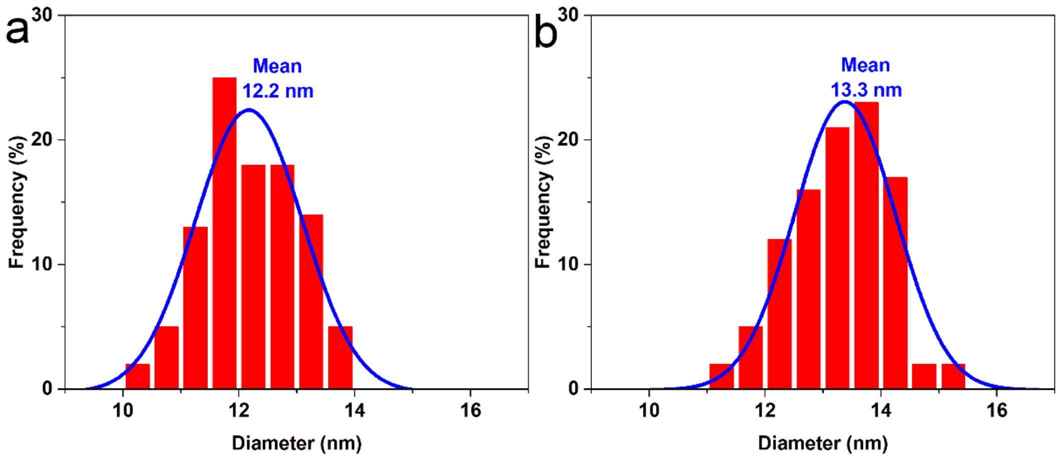

Figure S15. Size distribution of $NaYF_4$:80%Yb NCs: (a) primary sample, (b) sample annealed in OA/ODE.

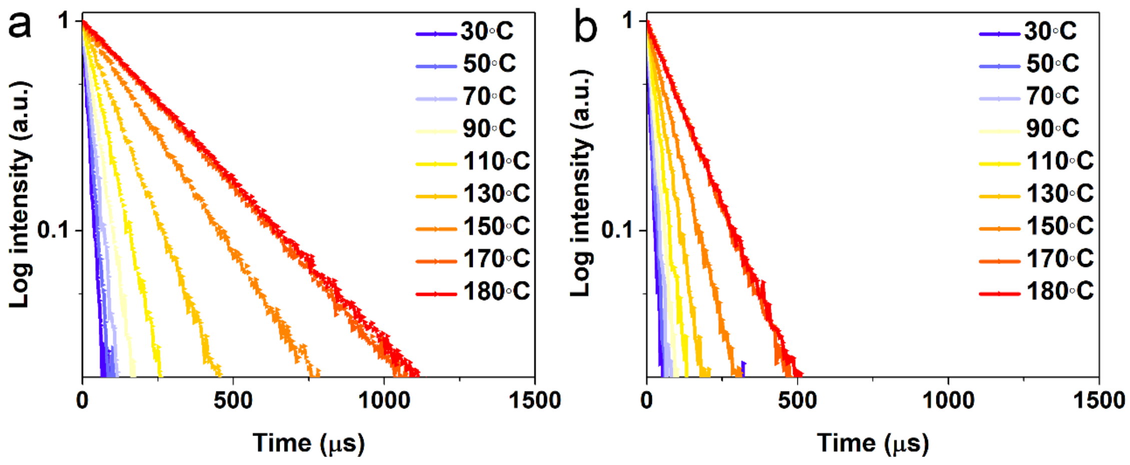

Figure S16. Yb^{3+} decay curves under elevated temperatures for the $NaYF_4$:80%Yb NCs before (a) and after (b) wet-chemistry annealing in OA and ODE solvents.

Table S4. Lifetime value comparison of $NaYF_4$:80%Yb nanocrystals before and after wet-chemistry annealing in OA and ODE solvents.

Temperature	30°C	50°C	70°C	90°C	110°C	130°C	150°C	170°C	180°C
Before annealing (μs)	17.2	22.8	29.1	43.0	67.5	114.5	199.0	275.7	285.6
After annealing (μs)	11.2	14.5	17.7	23.6	32.4	49.6	75.4	121.9	124.6
